# A randomised controlled trial of an Intervention to Improve Compliance with the ARRIVE guidelines (IICARus)

**DOI:** 10.1186/s41073-019-0069-3

**Published:** 2019-06-12

**Authors:** Kaitlyn Hair, Malcolm R. Macleod, Emily S. Sena, on behalf of the IICARus Collaboration, Emily S. Sena, Kaitlyn Hair, Malcolm R. Macleod, David Howells, Philip Bath, Cadi Irvine, Catriona MacCallum, Gavin Morrison, Alejandra Clark, Gina Alvino, Michelle Dohm, Jing Liao, Chris Sena, Rosie Moreland, Fala Cramond, Gillian L. Currie, Zsanett Bahor, Paula Grill, Alexandra Bannach-Brown, Daniel-Cosmin Marcu, Sarah Antar, Katrina Blazek, Timm Konold, Monica Dingwall, Victoria Hohendorf, Mona Hosh, Klara Zsofia Gerlei, Kimberley Elaine Wever, Victor Jones, Terence J. Quinn, Natasha A. Karp, Jennifer Freymann, Anthony Shek, Teja Gregorc, Arianna Rinaldi, Privjyot Jheeta, Ahmed Nazzal, David Ewart Henshall, Joanne Storey, Julija Baginskaite, Cilene Lino de Oliveira, Kamil Laban, Emmanuel Charbonney, Savannah A. Lynn, Marco Cascella, Emily Wheater, Daniel Baker, Ryan Cheyne, Edward Christopher, Paolo Roncon, Evandro Araújo De-Souza, Mahmoud Warda, Sarah Corke, Zeinab Ammar, Leigh O’Connor, Ian M. Devonshire, Sarah K. McCann, Laura J. Gray, Ezgi Tanriver-Ayder

**Affiliations:** Centre for Clinical Brain Sciences, University of Edinburgh, Edinburgh, UK

**Keywords:** ARRIVE, Reporting guidelines, Randomised controlled trial

## Abstract

**Background:**

The ARRIVE (Animal Research: Reporting of In Vivo Experiments) guidelines are widely endorsed but compliance is limited. We sought to determine whether journal-requested completion of an ARRIVE checklist improves full compliance with the guidelines.

**Methods:**

In a randomised controlled trial, manuscripts reporting in vivo animal research submitted to PLOS ONE (March–June 2015) were randomly allocated to either requested completion of an ARRIVE checklist or current standard practice. Authors, academic editors, and peer reviewers were blinded to group allocation. Trained reviewers performed outcome adjudication in duplicate by assessing manuscripts against an operationalised version of the ARRIVE guidelines that consists 108 items. Our primary outcome was the between-group differences in the proportion of manuscripts meeting all ARRIVE guideline checklist subitems.

**Results:**

We randomised 1689 manuscripts (control: *n* = 844, intervention: *n* = 845), of which 1269 were sent for peer review and 762 (control: *n* = 340; intervention: *n* = 332) accepted for publication. No manuscript in either group achieved full compliance with the ARRIVE checklist. Details of animal husbandry (ARRIVE subitem 9b) was the only subitem to show improvements in reporting, with the proportion of compliant manuscripts rising from 52.1 to 74.1% (*X*^2^ = 34.0, df = 1, *p* = 2.1 × 10^−7^) in the control and intervention groups, respectively.

**Conclusions:**

These results suggest that altering the editorial process to include requests for a completed ARRIVE checklist is not enough to improve compliance with the ARRIVE guidelines. Other approaches, such as more stringent editorial policies or a targeted approach on key quality items, may promote improvements in reporting.

**Electronic supplementary material:**

The online version of this article (10.1186/s41073-019-0069-3) contains supplementary material, which is available to authorized users.

## Background

There are widespread failures across in vivo animal research to adequately describe and report research methods, including critical measures to reduce the risk of experimental bias [10, 13]. Such omissions have been shown to be associated with overestimation of effect sizes [8, 13] and are likely to contribute, in part, to translational failure. In an effort to improve reporting standards, an expert working group coordinated by the National Centre for the Replacement, Refinement and Reduction of Animals in Research (NC3Rs) developed the Animal Research: Reporting of In Vivo Experiments (ARRIVE) guidelines [9], published in 2010.

Since the ARRIVE guidelines were first published, they have been endorsed by many journals in their instructions to authors, but this has not been accompanied by substantial improvements in reporting [2, 3, 6, 14]. Simply endorsing the guidelines does not appear to be sufficient to encourage compliance. Recent findings suggest that following the introduction of mandated completion of a distinct reporting checklist at ten Nature Journals at the stage of first revision significantly improved the quality in reporting versus that of comparator journals [7, 12].

PLOS ONE is an open access online only journal which at the time this study began published around 32,000 research articles per year. Of these, some 5000 were described in vivo animal research. At present, PLOS ONE instructions to authors encourage compliance with the ARRIVE guidelines, but do not mandate checklist completion. Journals have an important role to play in ensuring that the quality of reporting in the research they publish is robust, yet the most effective mechanism by which they can achieve this remains unclear.

Our aim was to assess the effect of an email request to authors to complete an ARRIVE checklist on compliance with the ARRIVE guidelines. The email request was at the time of submission and requested that authors state where in the manuscript various components of the ARRIVE guidelines are reported.

## Methods

### Methodology and open data

Our protocol, data analysis plan, analysis code, data validation code, and complete dataset are available on the Open Science Framework (10.17605/OSF.IO/XSJBV).

### Ethical approval

We sought an informal ethical opinion from the BMJ Ethics Committee, who were prepared to consider our proposal although it was slightly out of scope. We did this because we were unable at the time to identify an institutional ethics committee who considered this research to fall within their remit. The majority view of the committee was that it was ethical for manuscripts to be randomised between different handling methods; that it was ethical for authors, peer reviewers, and academic editors to be kept unaware of the existence of the study while it was in progress; and that it was ethical for the study to receive funding from the NC3Rs.

### Randomisation of manuscripts

We developed (10.5281/zenodo.1188821) an online platform to support each stage of the project (https://ecrf1.clinicaltrials.ed.ac.uk/iicarus/).

The PLOS ONE editorial process involves an initial screening process, including a determination of whether a manuscript describes animal studies, whether it describes human studies (one manuscript might describe both), and categorises the area of research according to an established taxonomy. For studies reporting the use of animals, checks are carried out to ensure that appropriate institutional animal care and use committee/ethical approvals were in place, and authors of studies perceived to be at high risk—for instance those animal studies which used death as an endpoint—are contacted to provide a valid justification. Manuscripts are then allocated to an academic editor (AE), who assigns peer reviewers as appropriate.

Manuscripts submitted to PLOS ONE between March and June 2015 describing in vivo animal research were randomised using the IICARus web platform to receive standard editorial processing (control group) or checklist completion requests (intervention group). The randomisation sequence was generated using randomisation in the C# programming language and involved minimisation (weighted at 0.75) to ensure that country of origin (of the corresponding author) was balanced between groups. All users of the platform were assigned a level of access in line with their role (e.g. “Trainee”, “Reviewer”, “Randomiser”) to ensure that any individual involved in outcome assessment was blinded to the allocation sequence.

On submission, authors receive an automated acknowledgement from the publisher that their submission had entered a screening phase. For manuscripts identified during screening to include in vivo research and which were randomised to the intervention, corresponding authors were informed in the post screening email that a completed ARRIVE checklist must be completed before the manuscript could advance through the review process. The email advised that this should include details of the page of their manuscript on which each ARRIVE item was addressed (an excerpt from the email used for the intervention is available on the OSF 10.17605/OSF.IO/XSJBV). If the PLOS editorial team did not receive a checklist, it was sent back to authors once more for completion. Manuscripts by authors who did not complete the checklist after the second contact, for any reason, were still passed to the next stage and continued in the study. The contents of completed checklists were not checked against the manuscript for compliance at any stage.

### Blinded manuscript processing

Authors, AEs, and peer reviewers were blinded to the existence of the study. Study personnel took care, in their public comments, not to disclose details of the study or the journal at which the study was being conducted. The journal was not named in the study protocol. If authors enquired as to why their manuscript was being processed differently, they were to be advised that these differences were due to variation within the editorial team in the intensity with which they pursued efforts to improve the review process.

For studies randomised to the control group, PLOS ONE processed the manuscript according to their normal editorial processes.

Once a final decision regarding publication was made, the pre-publication materials for accepted manuscripts were collated by the PLOS editorial team. Where an ARRIVE checklist was included in the accepted materials for publication, this was redacted, along with any reference in the text to the submission of a completed ARRIVE checklist. The format of manuscripts largely excluded any evidence that the manuscript was submitted to PLOS ONE. If a reference to PLOS ONE was discovered in the text by internal outcome assessors (within our research group), this was also redacted to prevent any change in behaviour which may result from external outcome assessors knowing which publisher was involved in the study. Where authors stated that the work complies with the ARRIVE guidelines this statement was not redacted. Redacted PDFs of all materials were provided to our research team and uploaded to the IICARus web platform.

### Outcome assessment

Primary, secondary, tertiary, and feasibility outcomes are confirmatory and were pre-specified in our study protocol (10.17605/OSF.IO/XSJBV). The unit of analysis for all outcomes was the manuscript.

Our primary outcome was to assess whether the proportion of publications in each group considered to fully comply with all of the ARRIVE criteria (at the level of the 38 subitems) was independent of group allocation.

To assess compliance with the ARRIVE guidelines in greater detail, we assessed reporting at the subitem level. The Landis core reporting standards [11] set out the key aspects of study design and conduct necessary to improve transparency of in vivo research and allow readers to ascertain the validity of the findings reported. We therefore wanted to assess compliance with the ARRIVE items which form these criteria, namely, reporting of randomisation to experimental groups (subitem 6b), reporting of blinded outcome assessment (subitem 6b), reporting of a sample size calculation (subitem 10a), and reporting of animal exclusions (subitem 15b). In addition, we aimed to investigate any effects our intervention may have on the process of publication by assessing the number of manuscripts accepted for publication in each group.

Our secondary outcomes were therefore to assess whether:

- The proportion of publications meeting each of the individual 38 ARRIVE subitems was independent of group allocation (intervention/ control)
- The proportion of studies reporting all Landis criteria subitems present in the ARRIVE guidelines was independent of group allocation
- The proportion of submitted manuscripts accepted for publication was independent of group allocation

We also wanted to examine whether different domains, countries, or research that includes human research may be associated with differences in adherence to the ARRIVE guidelines.

For our tertiary outcomes, we assessed whether:

- The proportion of publications meeting each of the 38 ARRIVE subitems was independent of group allocation, stratified by experimental animal
- The proportion of studies reporting all of the Landis criteria subitems (blinded assessment of outcome, sample size calculation, and criteria for exclusion of experimental subjects), stratified by experimental animal, was independent of group allocation
- The proportion of publications meeting each of the 38 ARRIVE subitems was independent of group allocation, stratified by the country of the address of the corresponding author
- The proportion of publications meeting each of the 38 ARRIVE subitems was independent of group allocation, stratified by whether or not the research also contains human data
- To examine the feasibility of implementing requests for ARRIVE checklist completion at PLOS ONE, we assessed outcomes relating to the duration of processing manuscripts to gain an insight into potential costs to the journal. We therefore assessed the following for accepted manuscripts in each group:
- Time (days) spent in PLOS editorial office in handling the manuscript (prior to editor assignment).
- Time (days) from manuscript submission to AE assignment.
- Time (days) from AE assignment to first reviewer agreed.
- Time (days) from AE assignment to first decision
- Time (days) from receipt of last review to AE decision

In addition, we assessed the following outcomes in manuscripts that were accepted following resubmission:

- Time (days) from initial decision letter to resubmission
- Number of cycles of resubmission
- Time (days) from resubmission to final decision

We also conducted some exploratory analyses not defined in the study protocol. Although the majority of authors complied with the request to complete an ARRIVE checklist, a small number did not. By limiting our analyses to the manuscripts that had received the intervention (equivalent to an “on treatment” analysis), we assessed:

- The proportion of publications meeting each of the 38 ARRIVE subitems in the control and “on treatment” intervention group

The Landis reporting criteria are minimal reporting standards which are important to quality improvement efforts. Given that the reporting of randomisation to experimental groups and blinded outcome assessment are part of the same subitem (6b) in the ARRIVE guidelines, we explored each of the Landis criteria items individually to assess:

- The proportion of publications meeting each individual Landis criteria in each group

We divided the 20 main ARRIVE items into 38 subitems. These subitems were further operationalised 108 questions (Additional file 1) which were scored by trained outcome assessors on the web platform. Two of these questions (0.1.0 *What animal species are used in this research?* and 0.2.0*: Does the manuscript include human study?*) were used only to categorise research for analysis purposes (Tertiary outcomes) and were not included in assessments of compliance. It was later determined by the steering committee that six questions from the original 108 were not strictly required to comply with the ARRIVE checklist (see Additional file 1; removed questions highlighted in grey) and were therefore also excluded from the analysis. The decision to remove data from these six questions was taken after data collection, but prior to the unblinding of group allocation.

PDF files of manuscripts were available alongside the scoring questions. Each manuscript was scored by two independent reviewers who were blinded to both intervention status and to the score given by the alternative reviewer. Manuscripts were presented to reviewers in random order, and the platform did not allow the same user to review the same manuscript twice. Discrepancies between reviewers were reconciled by a third independent reviewer, who could view both previous scores. For some manuscripts, some questions were not applicable (e.g. questions relating to fish studies in a manuscript describing rat experiments). Compliance was only assessed against questions that were applicable to the study.

There were several deviations from the outcome measures specified our study protocol. The time spent in the PLOS editorial office was not disentangled from time with the authors; therefore, it includes time for the authors to follow any copyediting changes and requests for documents (including the request to complete an ARRIVE checklist). Similarly, the time spent with authors was also included in the time from manuscript submission to AE assignment. In addition, we had originally intended to analyse the time in the PLOS editorial office in minutes, but the measurement of this was not feasible. We were unable to analyse “The proportion of submitted manuscripts accepted for publication, stratified by experimental animal” (Secondary outcome measure) as we did not receive species categorisation data for studies which were not accepted. PLOS ONE were unable to provide us with one of our specified feasibility measures “Time (days) for each reviewer, from solicitation of reviews to receipt of reviews)” and instead provided “Time (days) from AE assignment to first decision”. “Time (days) from AE assignment to first reviewer agreed” also differs to the outcome described in our study protocol (“Time (days) from AE assignment to solicitation of reviews”). In addition, we had originally set out in our protocol that we would look at the following feasibility outcome measures for manuscripts when the decision was other than “Accept” or “Reject”: whether a revised manuscript is submitted, time (days) from initial decision letter to resubmission, number of cycles of resubmission, and time (days) from resubmission to final decision. However, we did not attain this information for manuscripts which were not eventually accepted. Therefore, all feasibility measures apply to accepted manuscripts only.

### Reviewer training

This was a challenging project, and we used crowdsourcing to recruit additional reviewers external to our research group. We used our research networks and social media to identify researchers and students across the biomedical sciences and recruit them as outcome assessors for the project. As an incentive, rewards were given to external reviewers who reached a pre-specified number of manuscript reviews or completed the most reviews in a certain time period.

To ensure that review quality was high, we required reviewers to complete online training prior to reviewing manuscripts as part of the project. We developed a training program with a pool of 10 manuscripts for which we described “Gold standard” correct answers with explanations and an accompanying document with further elaboration (10.17605/OSF.IO/XSJBV). To successfully complete the training, external reviewers had to score 80% against these gold standard answers overall and score 100% on gold standard questions relating to the Landis criteria subitems for three consecutive training manuscripts. The training platform remains available (https://ecrf1.clinicaltrials.ed.ac.uk/iicarus/) and can still be used as a training tool for assessing manuscripts against the ARRIVE guidelines.

### Power calculations

When the study was being designed, the PLOS ONE editorial team estimated that complete compliance with the ARRIVE guidelines was close to zero. To have 80% power with an alpha of 0.05, to detect an increase in full compliance from 1 to 10% (the primary outcome) would require 100 published manuscripts per group. To examine each of the individual 38 ARRIVE subitems (Secondary outcome), after correction for multiplicity of testing (alpha = 0.0013), we would require 200 published manuscripts per group to detect with 80% power an increase from 30 to 50% in the prevalence of reporting of an individual subitem. It was estimated that at time of the trial PLOS ONE accepted around 70% of manuscripts, and to account for some drop out because of the use of the same academic editor, we increased our group estimate to 150 manuscripts per group for the primary outcome, and 300 manuscripts in each group for secondary outcomes. During the course of the study, it appeared that acceptance rates were lower than the estimate, and so we increased target recruitment to 1000 manuscripts, of which we estimated 600 would be accepted for publication. Despite the risk of overpowering, we did not curtail the study when we had reached the required number of manuscripts accepted for publication because we were concerned that manuscripts with short submission to acceptance times would be enriched in the study population and might not be representative of all manuscripts.

### Data validation

We validated the dataset, blinded to group allocation, to minimise errors. For example, where a manuscript was assessed as not including fish experiments, “Yes” or “No” responses to IICARus questions only relevant to fish species (e.g. Additional file 1, Questions 9.2.3–9.2.4) should not have been recorded. The R code for validation, with explanations of each response validated, and the changes we made to the data are available on the OSF. These were uploaded prior to the unblinding of the final results, at which point database lock occurred and the data were not subsequently altered in any way.

### Statistical analysis

All analyses were carried out using RStudio v1.0.143 with the level of statistical significance set at *p* < 0.05, corrected as appropriate for multiple comparisons. Our full statistical analysis plan and accompanying R code was uploaded to the OSF prior to database lock.

We performed logistic regression with group allocation and corresponding author country of origin included as independent variables to determine any effects on full compliance (primary outcome) and compliance with each of *the* 38 subitems (secondary outcome), adjusting for the minimised randomisation. For our primary, secondary, and tertiary outcome measures, we used the chi-squared test of independence to test whether compliance was independent of group membership (intervention/control). To determine if the differences between proportions is meaningful for each outcome measure, effect sizes were calculated using Cohen’s H. For feasibility outcomes, medians and interquartile ranges were calculated and the Mann-Whitney *U* test was used to test whether a significant difference existed between groups. To control the familywise error rate of multiple comparisons, the Holm-Bonferroni method [1] was used to adjust *p* values for secondary, tertiary, and feasibility outcomes. Only means and confidence intervals were calculated for our exploratory analyses. A full description of our data analysis plan is available on the OSF: https://osf.io/zgqxk/.

### Statistical considerations

The proportion of compliant manuscripts was assessed based on the number compliant manuscripts divided by the number of applicable manuscripts. In some cases, particularly when stratifying by manuscript country of origin or animal species, the number of manuscripts in each group is very low. If the number of applicable manuscripts for any subitem (with or without stratification) was less than 10, we did not perform statistical analysis.

The chi-squared test of Independence relies on the assumption that no more than 20% of expected counts are less than 5 and that no individual expected counts are less than 1. In cases where counts were less than 5, a Fisher’s exact test was used.

Unpaired *t* tests rely on a normal distribution; therefore, if the distribution was non-normal, the Mann-Whitney *U* test, a non-parametric alternative, was used and summary medians and interquartile ranges were presented. In the case of parametric data with unequal variance between groups, Welch’s *t* test was used due to higher reliability.

## Results

**Fig. 1:**
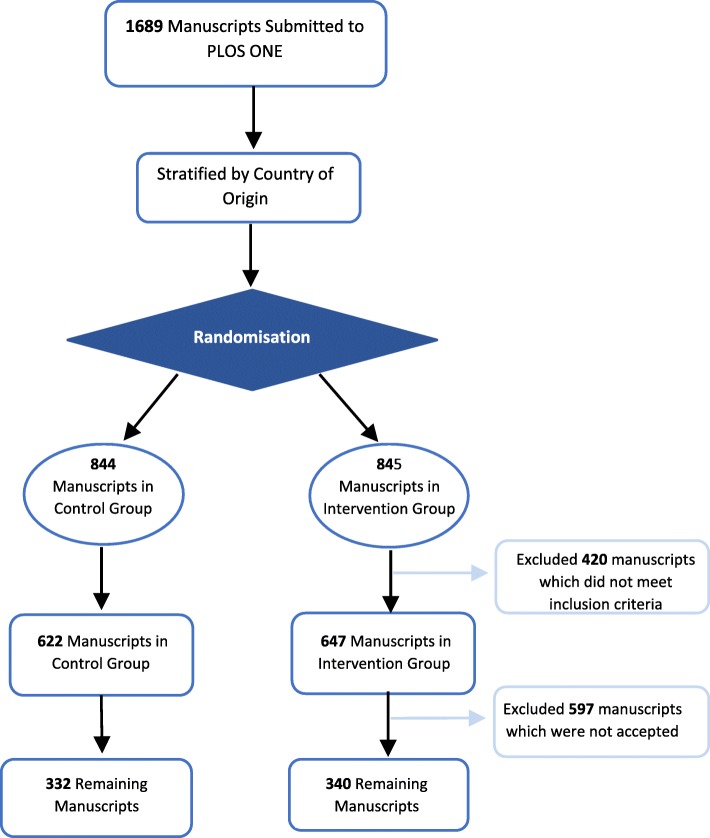
Manuscript processing

We randomised 1689 PLOS ONE manuscripts: 845 manuscripts to the intervention and 844 to control. Of these, 192 were rejected by the journal and 75 manuscripts were withdrawn. A further 153 were excluded due to errors, including 77 that were found not to contain in vivo animal research. The reasons for these errors are shown in Additional file 2. Of the remaining 1269 manuscripts, 672 were accepted for publication (340 control, 332 intervention) and underwent web-based outcome assessment (Fig. 1). Manuscript allocation to group and the corresponding number of manuscripts from each country which were randomised and accepted are shown in Table 1. No authors questioned the differences in manuscript processing occurring within the intervention group. A complete dataset detailing the proportion compliance for each of the 108 questions is available online (10.17605/OSF.IO/XSJBV).

**Table 1:** Manuscript allocation by country. Manuscripts allocated to each group per corresponding author country of origin; nR, number randomised; nA, number accepted

	Control		Intervention			Control		Intervention	
Country	nR	nA	nR	nA	Country	nR	nA	nR	nA
Algeria	1	0	0	0	Malaysia	2	0	2	1
Argentina	4	1	3	1	Mexico	4	1	3	2
Australia	13	6	13	11	Netherlands	9	6	13	12
Austria	6	3	1	1	North Korea	5	4	0	0
Belgium	3	3	4	4	New Zealand	0	0	1	1
Brazil	29	13	33	13	Norway	3	3	0	0
Canada	15	12	16	12	Pakistan	0	0	1	0
Chile	1	1	4	2	Poland	2	2	5	3
China	135	38	157	54	Portugal	2	1	6	3
Colombia	1	1	0	0	Puerto Rico	0	0	1	0
Czech Republic	1	1	1	0	Romania	1	1	1	0
Denmark	6	3	8	3	Russia	3	2	1	1
Egypt	2	1	6	4	Saudi Arabia	3	2	1	0
Finland	1	0	1	1	Singapore	3	1	7	2
France	10	6	15	13	Slovakia	1	0	0	0
French Guiana	1	0	0	0	South Africa	1	1	2	0
Germany	34	24	29	13	South Korea	28	14	26	8
Greece	2	2	0	0	Spain	15	8	11	7
Hong Kong	1	0	3	2	Sweden	10	7	11	6
Hungary	1	0	0	0	Switzerland	6	5	7	5
India	15	8	10	3	Taiwan	19	13	8	3
Iran	1	1	1	1	Thailand	0	0	1	0
Ireland	2	2	1	1	Turkey	2	0	1	0
Israel	1	1	2	2	Ukraine	0	0	1	0
Italy	8	6	15	11	United Kingdom	17	10	18	9
Japan	47	27	46	23	United States	143	97	150	94
Kuwait	2	2	0	0					

### Quality of outcome assessment

Three hundred sixty individuals registered with the online platform; 47 completed reviewer training, and 42 contributed at least one outcome assessment. The percentage agreement between the first and second reviewer for each manuscript was high. For 71.6% (481/672) of manuscripts, reviewers were in agreement on at least 80% of the questions. The agreement of reviewers varied considerably at the level of each of the 108 individual questions (Additional file 3: Table S1), from a kappa coefficient of 0.90 (0.86–0.93) for Question 1.1 *(Is the species of animal model studied reported in the title?*) to a worse than chance kappa coefficient of − 0.03 (− 0.10 - -0.04) for Question 13.2 *(Is the unit of analysis for at least one test explicitly specified?)*. This distribution of kappa agreement is displayed in a histogram (Additional file 3: Fig. S1).

### Primary outcome

No manuscript achieved full compliance with the ARRIVE checklist; therefore, there was no difference between the control and intervention groups (*X*^2^ = 0.1, df = 1, *p* = 0.76). Compliance with individual ARRIVE subitems ranged from 8 to 65%. The median compliance was 36.8% and 39.5% of relevant subitems in the control and intervention groups, respectively.

### Logistic regression

Country of corresponding author had no influence on compliance either overall or for any individual subitems. Only one subitem had improved reporting in the intervention group versus control, subitem 9b (*Provide details of husbandry conditions* e.g. *breeding programme, light/dark cycle, temperature, quality of water etc for fish, type of food, access to food and water, environmental enrichment*) (increased log odds of compliance by 1.03 (*p* < 0.0001).

### Secondary outcomes

#### Compliance with individual ARRIVE subitems

**Fig. 2:**
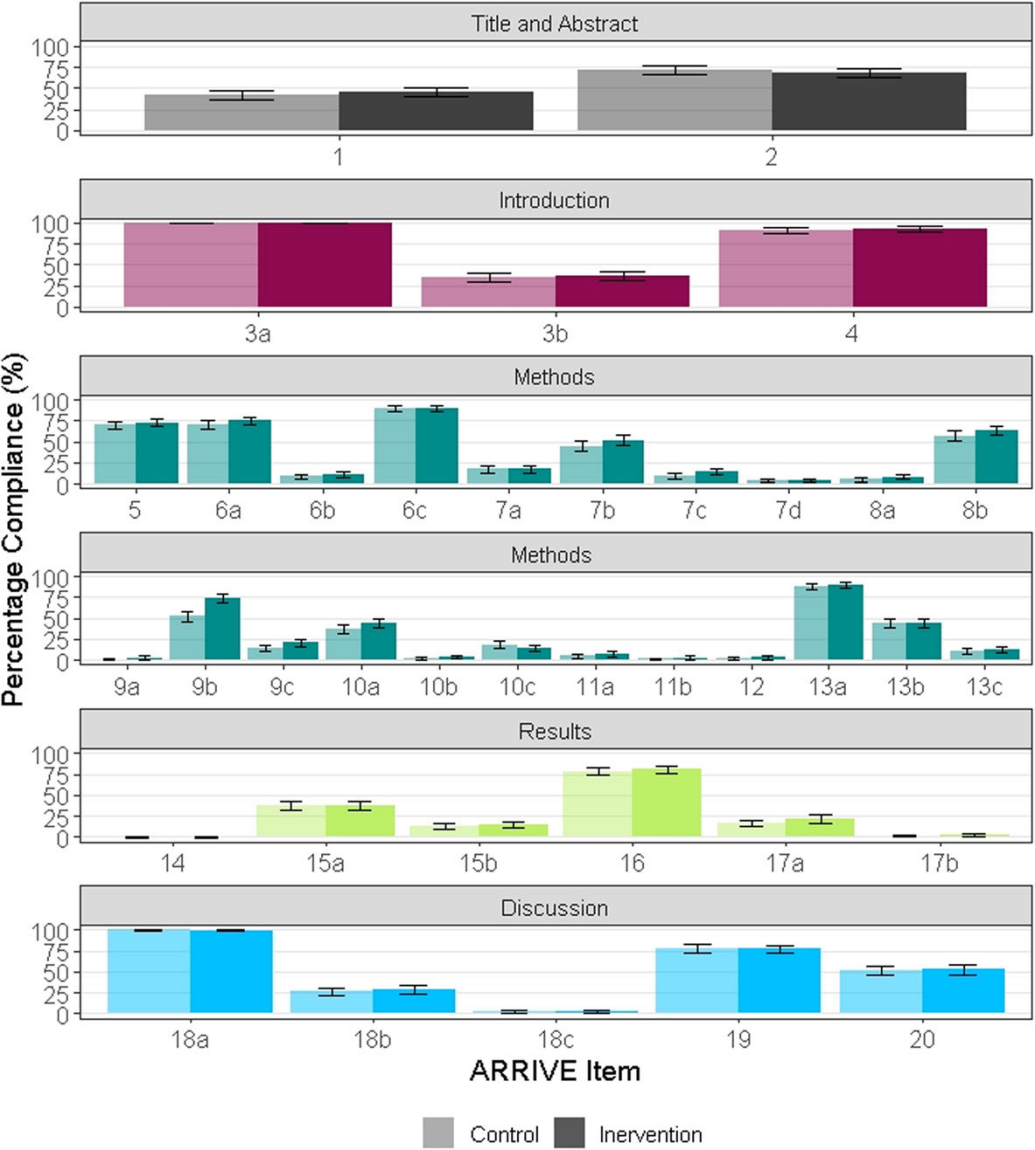
Percentage compliance for each ARRIVE subitem; percentage compliance for each ARRIVE subitem with 95% confidence intervals; asterisk denotes statistical significance; figure divided into article sections specified in the ARRIVE guidelines

Only one ARRIVE subitem had significantly improved compliance in the intervention group (Table 2). ARRIVE subitem 9b (*Provide details of husbandry conditions* e.g. *breeding programme, light/dark cycle, temperature, quality of water etc for fish, type of food, access to food and water, environmental enrichment*) was reported in 52.1% (177/340) of manuscripts in the control group compared to 74.1% (246/332) of manuscripts in the intervention group (*X*^2^ = 34.0, df = 1, *p* < 0.0001). Reporting of animal characteristics and health status (subitem 14) was very low, with 0.29% (1/339) and 0% (0/332) compliance in the control and intervention groups, respectively. Similarly, reporting of animal housing (subitem 9a); adverse events (subitem 17b); the order of treatment and assessment (Item 11b); implications for replacement, refinement, or reduction (subitem 18c); defining primary and secondary outcomes (subitem 12); and rationale for experimental procedures (subitem 7d) was low, with less than 5% of manuscripts reporting each of these items in both groups. Figure 2 shows the percentage compliance in each group for each ARRIVE subitem in each section of the manuscript.

**Table 2:** Percentage compliance for each ARRIVE subitem; %, percentage of compliant manuscripts; CI, confidence interval; *n*, number of compliant manuscripts; *N*, total number of applicable manuscripts; Adj *p*, adjusted *p* value; Cohen’s H, Cohen’s H effect size

	Control				Intervention					
ARRIVE subitem	%	95% CIs	*n*	*N*	%	95% CIs	*n*	*N*	Adj. *p*	Cohen’s H
1	41.76	36.5–47.2	142	340	44.58	39.2–50.1	148	332	> 0.99	0.06
2	71.76	66.6–76.4	244	340	67.47	62.1–72.4	224	332	> 0.99	−0.09
3a	100.00	98.6–100	340	340	100.00	98.6–100	332	332	> 0.99	0.00
3b	34.12	29.1–39.5	116	340	36.14	31–41.6	120	332	> 0.99	0.04
4	91.18	87.5–93.9	310	340	93.07	89.6–95.5	309	332	> 0.99	0.07
5	69.41	64.2–74.2	236	340	72.59	67.4–77.3	241	332	> 0.99	0.07
6a	70.00	64.8–74.8	238	340	75.00	69.9–79.5	249	332	> 0.99	0.11
6b	8.33	5.7–12	28	336	10.49	7.5–14.5	34	324	> 0.99	0.07
6c	90.00	86.2–92.9	306	340	88.86	84.8–91.9	295	332	> 0.99	−0.04
7a	16.76	13–21.3	57	340	16.87	13.1–21.4	56	332	> 0.99	0.00
7b	44.37	38.7–50.2	134	302	51.33	45.5–57.1	154	300	> 0.99	0.14
7c	8.64	5.8–12.5	26	301	14.09	10.5–18.7	42	298	> 0.99	0.17
7d	3.63	1.9–6.6	11	303	3.63	1.9–6.6	11	303	> 0.99	0.00
8a	4.71	2.8–7.7	16	340	7.83	5.3–11.4	26	332	> 0.99	0.13
8b	57.06	51.6–62.4	194	340	62.65	57.2–67.8	208	332	> 0.99	0.11
9a	0.30	0–1.9	1	337	2.74	1.3–5.3	9	328	0.39	0.22
9b	52.06	46.6–57.5	177	340	74.10	69–78.7	246	332	< 0.001	0.46
9c	14.71	11.2–19	50	340	20.48	16.4–25.3	68	332	> 0.99	0.15
10a	37.35	32.2–42.8	127	340	43.67	38.3–49.2	145	332	> 0.99	0.13
10b	3.53	1.9–6.2	12	340	7.53	5–11.1	25	332	> 0.99	0.18
10c	18.15	14.3–22.8	61	336	14.64	11.1–19.1	47	321	> 0.99	−0.10
11a	4.82	2.8–8	15	311	7.49	4.9–11.2	23	307	> 0.99	0.11
11b	1.24	0.4–3.4	4	323	2.88	1.4–5.6	9	313	> 0.99	0.12
12	1.76	0.7–4	6	340	3.01	1.5–5.6	10	332	> 0.99	0.08
13a	87.50	83.4–90.7	294	336	89.91	86–92.9	294	327	> 0.99	0.08
13b	44.08	38.7–49.6	149	338	44.51	39.1–50.1	146	328	> 0.99	0.01
13c	10.06	7.2–13.9	34	338	12.80	9.5–17	42	328	> 0.99	0.09
14	0.29	0–1.9	1	340	0.00	0–1.4	0	332	> 0.99	−0.11
15a	37.35	32.2–42.8	127	340	37.35	32.2–42.8	124	332	> 0.99	0.00
15b	12.65	9.4–16.8	43	340	14.46	10.9–18.8	48	332	> 0.99	0.05
16	78.55	73.7–82.8	260	331	80.94	76.1–85	259	320	> 0.99	0.06
17a	16.47	12.8–20.9	56	340	21.69	17.5–26.6	72	332	> 0.99	0.13
17b	1.18	0.4–3.2	4	340	1.81	0.7–4.1	6	332	> 0.99	0.05
18a	100.00	98.6–100	340	340	99.40	97.6–99.9	330	332	> 0.99	−0.16
18b	26.47	21.9–31.6	90	340	28.31	23.6–33.5	94	332	> 0.99	0.04
18c	2.94	1.5–5.5	10	340	3.01	1.5–5.6	10	332	> 0.99	0.00
19	77.94	73.1–82.2	265	340	77.71	72.8–82	258	332	> 0.99	−0.01
20	51.47	46–56.9	175	340	52.71	47.2–58.2	175	332	> 0.99	0.02

#### Reporting of Landis 4 subitems

Reporting of the Landis 4 criteria (blinding, randomisation, animal exclusions, and use of a sample size calculation) was low and did not differ significantly between groups (Fisher’s estimate for difference = 0.61, df = 1, *p* = 0.73). 1.5% (5/340) of the control group manuscripts) and 0.9% (3/332) of intervention group manuscripts reported all four subitems of the Landis criteria.

#### Manuscript acceptance

There was no significant difference in the proportion of accepted manuscripts between the control and intervention groups, being 54.7% (340/622) and 51.3% (322/647) respectively. (*X*^2^ = 1.30, df = 1, *p* = 0.25).

### Tertiary outcomes

#### Compliance by animal species

We removed animal species from the analysis where fewer than ten manuscripts reported the use of a species for control and intervention groups, leaving only rat and mouse studies. In studies involving mice, only reporting of ARRIVE subitem, 9b (*Provide details of husbandry conditions* e.g. *breeding programme, light/dark cycle, temperature, quality of water etc for fish, type of food, access to food and water, environmental enrichment*) increased significantly from 49.5% (105/211) in the control group to 70.2% (135/192) in the intervention group (*X*^2^ = 16.8, df = 1, *p* = 0.003). No subitem had a statistically significant difference between groups in rat studies. Results are summarised in Tables 3 and 4. There was no difference in Landis 4 compliance between animal species.

**Table 3:** ARRIVE subitem compliance in mouse studies. %, percentage of compliant manuscripts; CI, confidence interval; *n*, number of compliant manuscripts; *N*, total number of applicable manuscripts; Adj *p*, adjusted *p* value; Cohen’s H, Cohen’s H effect size

	Control				Intervention					
ARRIVE subitem	%	95% CIs	*n*	*N*	%	95% CIs	*n*	*N*	Adj *p*	Cohen’s H
1	37.44	31–44.4	79	211	37.50	30.7–44.8	72	192	> 0.99	0.00
2	68.25	61.4–74.4	144	211	60.42	53.1–67.3	116	192	> 0.99	− 0.16
3a	100.00	97.8–100	211	211	100.00	97.6–100	192	192	> 0.99	0.00
3b	30.33	24.3–37.1	64	211	29.69	23.4–36.8	57	192	> 0.99	− 0.01
4	89.10	83.9–92.8	188	211	92.19	87.2–95.4	177	192	> 0.99	0.11
5	67.77	61–73.9	143	211	71.88	64.9–78	138	192	> 0.99	0.09
6a	63.98	57.1–70.4	135	211	71.35	64.3–77.5	137	192	> 0.99	0.16
6b	5.24	2.8–9.4	11	210	7.89	4.6–12.9	15	190	> 0.99	0.11
6c	90.05	85–93.6	190	211	91.15	86–94.6	175	192	> 0.99	0.04
7a	17.54	12.8–23.5	37	211	17.71	12.7–24	34	192	> 0.99	0.00
7b	44.68	37.5–52.1	84	188	48.57	41–56.2	85	175	> 0.99	0.08
7c	6.91	3.9–11.8	13	188	9.83	6–15.5	17	173	> 0.99	0.11
7d	3.16	1.3–7.1	6	190	2.27	0.7–6.1	4	176	> 0.99	− 0.05
8a	4.74	2.4–8.8	10	211	6.25	3.4–10.9	12	192	> 0.99	0.07
8b	55.45	48.5–62.2	117	211	60.94	53.6–67.8	117	192	> 0.99	0.11
9a	0.47	0–3	1	211	2.08	0.7–5.6	4	192	> 0.99	0.15
9b	49.76	42.8–56.7	105	211	70.31	63.2–76.6	135	192	0.003	0.42
9c	15.17	10.7–20.9	32	211	23.96	18.2–30.7	46	192	> 0.99	0.22
10a	27.01	21.3–33.6	57	211	31.25	24.9–38.4	60	192	> 0.99	0.09
10b	2.84	1.2–6.4	6	211	5.73	3–10.3	11	192	> 0.99	0.14
10c	21.15	15.9–27.5	44	208	15.43	10.7–21.6	29	188	> 0.99	− 0.15
11a	4.12	1.9–8.3	8	194	5.00	2.5–9.6	9	180	> 0.99	0.04
11b	1.93	0.6–5.2	4	207	0.54	0–3.4	1	185	> 0.99	−0.13
12	0.00	0–2.2	0	211	3.65	1.6–7.7	7	192	0.40	0.38
13a	87.20	81.8–91.3	184	211	89.58	84.2–93.4	172	192	> 0.99	0.07
13b	46.92	40.1–53.9	99	211	42.71	35.7–50	82	192	> 0.99	− 0.08
13c	8.06	4.9–12.8	17	211	11.46	7.5–17	22	192	> 0.99	0.12
14	0.00	0–2.2	0	211	0.00	0–2.4	0	192	> 0.99	0.00
15a	36.02	29.6–42.9	76	211	36.46	29.7–43.7	70	192	> 0.99	0.01
15b	11.37	7.6–16.6	24	211	10.94	7.1–16.4	21	192	> 0.99	− 0.01
16	84.62	78.8–89.1	176	208	80.95	74.5–86.1	153	189	> 0.99	− 0.10
17a	15.64	11.2–21.4	33	211	23.44	17.8–30.2	45	192	> 0.99	0.20
17b	0.95	0.2–3.7	2	211	2.60	1–6.3	5	192	> 0.99	0.13
18a	100.00	97.8–100	211	211	98.96	95.9–99.8	190	192	> 0.99	− 0.20
18b	27.01	21.3–33.6	57	211	26.56	20.6–33.5	51	192	> 0.99	− 0.01
18c	3.32	1.5–7	7	211	3.13	1.3–7	6	192	> 0.99	− 0.01
19	82.46	76.5–87.2	174	211	81.77	75.4–86.8	157	192	> 0.99	− 0.02
20	51.18	44.2–58.1	108	211	56.25	48.9–63.3	108	192	> 0.99	0.10

**Table 4:** ARRIVE subitem compliance in rat studies. %, percentage of compliant manuscripts; CI, confidence interval; *n*, number of compliant manuscripts; *N*, total number of applicable manuscripts; Adj *p*, adjusted *p* value; Cohen’s H, Cohen’s H effect size

	Control				Intervention					
ARRIVE subitem	%	95% CIs	*n*	*N*	%	95% CIs	*n*	*N*	Adj *p*	Cohen’s H
1	47.27	33.9–61.1	26	55	59.09	46.3–70.8	39	66	> 0.99	0.24
2	83.64	70.7–91.8	46	55	81.82	70–89.9	54	66	> 0.99	− 0.05
3a	100.00	91.9–100	55	55	100.00	93.1–100	66	66	> 0.99	0.00
3b	20.00	10.9–33.4	11	55	34.85	23.8–47.7	23	66	> 0.99	0.34
4	96.36	86.4–99.4	53	55	96.97	88.5–99.5	64	66	> 0.99	0.03
5	83.64	70.7–91.8	46	55	80.30	68.3–88.7	53	66	> 0.99	− 0.09
6a	90.91	79.3–96.6	50	55	87.88	77–94.3	58	66	> 0.99	− 0.10
6b	24.53	14.2–38.6	13	53	21.54	12.7–33.8	14	65	> 0.99	− 0.07
6c	98.18	89–99.9	54	55	89.39	78.8–95.3	59	66	> 0.99	− 0.39
7a	9.09	3.4–20.7	5	55	12.12	5.7–23	8	66	> 0.99	0.10
7b	44.44	31.2–58.5	24	54	60.32	47.2–72.2	38	63	> 0.99	0.32
7c	5.56	1.4–16.3	3	54	14.29	7.1–25.9	9	63	> 0.99	0.30
7d	1.85	0.1–11.2	1	54	6.35	2.1–16.3	4	63	> 0.99	0.24
8a	1.82	0.1–11	1	55	10.61	4.7–21.2	7	66	> 0.99	0.39
8b	63.64	49.5–75.9	35	55	75.76	63.4–85.1	50	66	> 0.99	0.26
9a	0.00	0–8.1	0	55	6.15	2–15.8	4	65	> 0.99	0.50
9b	69.09	55–80.5	38	55	90.91	80.6–96.3	60	66	0.37	0.57
9c	12.73	5.7–25.1	7	55	13.64	6.8–24.8	9	66	> 0.99	0.03
10a	52.73	38.9–66.1	29	55	60.61	47.8–72.2	40	66	> 0.99	0.16
10b	1.82	0.1–11	1	55	12.12	5.7–23	8	66	> 0.99	0.44
10c	5.45	1.4–16.1	3	55	3.17	0.6–12	2	63	> 0.99	− 0.11
11a	3.77	0.7–14.1	2	53	10.77	4.8–21.5	7	65	> 0.99	0.28
11b	0.00	0–8.4	0	53	4.69	1.2–14	3	64	> 0.99	0.44
12	1.82	0.1–11	1	55	1.52	0.1–9.3	1	66	> 0.99	− 0.02
13a	94.55	83.9–98.6	52	55	90.77	80.3–96.2	59	65	> 0.99	− 0.15
13b	41.82	28.9–55.9	23	55	48.48	36.1–61	32	66	> 0.99	0.13
13c	18.18	9.5–31.4	10	55	16.67	9–28.3	11	66	> 0.99	− 0.04
14	0.00	0–8.1	0	55	0.00	0–6.9	0	66	> 0.99	0.00
15a	43.64	30.6–57.6	24	55	43.94	31.9–56.7	29	66	> 0.99	0.01
15b	12.73	5.7–25.1	7	55	16.67	9–28.3	11	66	> 0.99	0.11
16	70.37	56.2–81.6	38	54	87.30	76–94	55	63	> 0.99	0.42
17a	12.73	5.7–25.1	7	55	12.12	5.7–23	8	66	> 0.99	− 0.02
17b	1.82	0.1–11	1	55	0.00	0–6.9	0	66	> 0.99	− 0.27
18a	100.00	91.9–100	55	55	100.00	93.1–100	66	66	> 0.99	0.00
18b	18.18	9.5–31.4	10	55	28.79	18.6–41.4	19	66	> 0.99	0.25
18c	3.64	0.6–13.6	2	55	1.52	0.1–9.3	1	66	> 0.99	− 0.14
19	74.55	60.7–84.9	41	55	86.36	75.2–93.2	57	66	> 0.99	0.30
20	43.64	30.6–57.6	24	55	39.39	27.8–52.2	26	66	> 0.99	− 0.09

#### Feasibility measures

Re-assignment of academic editors occurred in a small number of cases (7/672), which confounds the recorded time in each stage and prevented us from analysing the feasibility outcomes for these manuscripts. The time from receipt of last review to final AE decision was missing from a large proportion of the remaining manuscripts (342/665), and so this analysis was not performed. Ten additional manuscripts were also excluded from the feasibility dataset due to missing data on one or more feasibility outcome s. After these exclusions, the feasibility analysis was performed on 328/340 manuscripts in the control group and 327/332 in the intervention group. For analysis of resubmitted articles, seven manuscripts were removed as these were accepted at first decision leaving 323/340 in the control group and 325/332 in the intervention group.

Upon examining histograms for each variable and calculating skew using the skew() function, all data for these outcomes were found to be right skewed, so we used Mann-Whitney *U* test to compare timings between groups. Time spent in the PLOS editorial office was significantly higher (*p* < 0.0001) for manuscripts in the intervention group with a median of 9 days (interquartile range [IQR] = 6–16.5) compared to the control group with a median of 6 days [3–10]. Time from submission to academic editor assignment was also significantly higher in the intervention group (13 days, range 9–22) than in the control group (9 days, range 7–14) (*p* < 0.0001). No statistically significant differences were identified for other feasibility outcomes (Table 5).

**Table 5:** Feasibility measures; Q1–Q3, interquartile range; *N*, number of applicable manuscripts; Adj *p*, adjusted p value; n.s, not significant

Feasibility outcomes	Control			Intervention			
	Median	Q1–Q3	*N*	Median	Q1–Q3	*N*	Adj *p*
Days in PLOS editorial office	6	3–10	328	9	6–16.5	327	< 0.0001
Days from submission to AE assignment	9	7–14	328	13	9–22	327	< 0.0001
Days from AE assignment to reviewer assignment	3	1–8	328	3	1–9	327	> 0.99
Days from AE assignment first decision	28	20–41.3	328	27	19–41	327	> 0.99
Days from initial decision to resubmission	41	23.5–51.5	323	40	23–45	325	> 0.99
Cycles of resubmission	1	1–2	323	1	1–2	325	> 0.99
Days from resubmission to final decision	31	15.5–58	323	34	16–59	325	> 0.99

#### Compliance by country

**Fig. 3:**
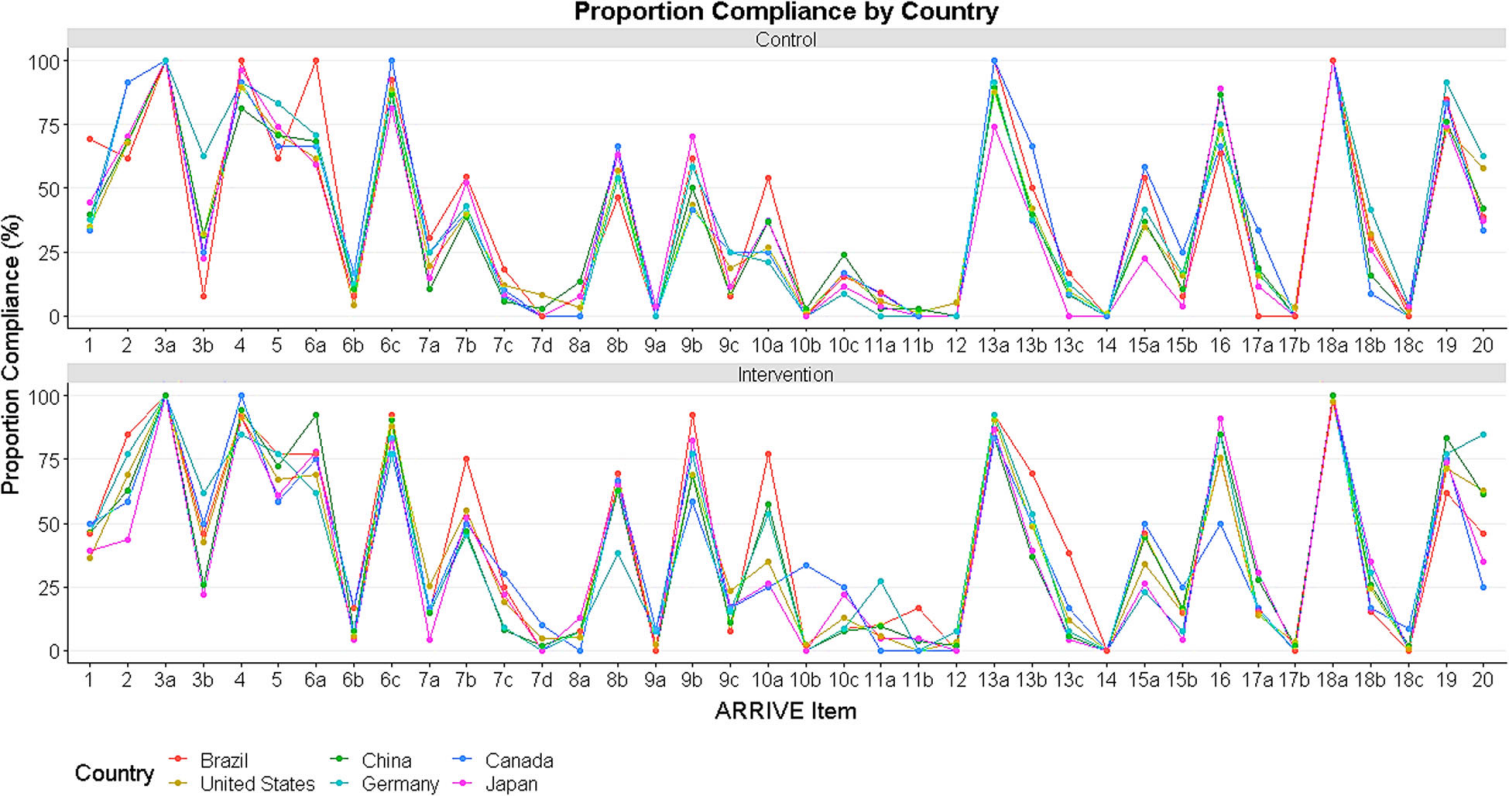
Compliance by country; percentage compliance for each ARRIVE subitem for manuscripts in each country (for countries with *N* manuscripts ≥10)

There were no statistically significant differences in compliance between control and intervention groups across any corresponding author country of origin. Although we did not set out to compare differences in compliance with different ARRIVE subitems across countries, we present these data in Fig. 3.

#### Human studies compliance

In manuscripts without human subjects, reporting of one ARRIVE subitem, 9b (*Provide details of husbandry conditions* e.g. *breeding programme, light/dark cycle, temperature, quality of water etc for fish, type of food, access to food and water, environmental enrichment*) increased significantly from 52.4% (172/316) in the control group to 76.9% (227/295) in the intervention group (*X*^2^ = 33.2, df = 1, *p* < 0.0001). In manuscripts containing human subjects, compliance also rose from 20.8% (5/24) to 51.35% (19/37) in the intervention group for this subitem, although we were limited by small sample sizes and this change was not found to be statistically significant (*X*^2^ = 4.47, df = 1, *p* = 1).

### Exploratory outcomes

#### Compliance in true intervention group

Despite allocation to the intervention group, a small subset (*n* = 31/332) of authors did not comply with the request to submit a completed checklist, and therefore, 31 manuscripts were in the intervention group without a completed ARRIVE checklist. We sought to determine compliance with each of the 38 subitems in the “true” intervention group (those submitted with a completed checklist), compared to the control group. The pattern of compliance is similar to that of the full intervention group compared to controls, suggesting that these instances of non-compliance did not impact on results. Summary statistics are presented in Table 6.

**Table 6:** True intervention ARRIVE subitem compliance; %, percentage of compliant manuscripts; CI, confidence interval; *n*, number of compliant manuscripts; *N*, total number of applicable manuscripts

	Control				Intervention			
ARRIVE subitem	%	95% CIs	*n*	*N*	%	95% CIs	*n*	*N*
1	41.76	36.5–47.2	142	340	45.85	40.1–51.7	138	301
2	71.76	66.6–76.4	244	340	66.45	60.8–71.7	200	301
3a	100.00	98.6–100	340	340	100.00	98.4–100	301	301
3b	34.12	29.1–39.5	116	340	35.88	30.5–41.6	108	301
4	91.18	87.5–93.9	310	340	93.02	89.4–95.5	280	301
5	69.41	64.2–74.2	236	340	72.43	66.9–77.3	218	301
6a	70.00	64.8–74.8	238	340	75.42	70.1–80.1	227	301
6b	8.33	5.7–12	28	336	9.49	6.5–13.6	28	295
6c	90.00	86.2–92.9	306	340	88.70	84.4–91.9	267	301
7a	16.76	13–21.3	57	340	16.94	13–21.8	51	301
7b	44.37	38.7–50.2	134	302	52.21	46.1–58.3	142	272
7c	8.64	5.8–12.5	26	301	14.07	10.3–18.9	38	270
7d	3.63	1.9–6.6	11	303	4.00	2.1–7.2	11	275
8a	4.71	2.8–7.7	16	340	7.97	5.3–11.8	24	301
8b	57.06	51.6–62.4	194	340	62.46	56.7–67.9	188	301
9a	0.30	0–1.9	1	337	3.03	1.5–5.9	9	297
9b	52.06	46.6–57.5	177	340	74.75	69.4–79.5	225	301
9c	14.71	11.2–19	50	340	21.26	16.9–26.4	64	301
10a	37.35	32.2–42.8	127	340	43.19	37.6–49	130	301
10b	3.53	1.9–6.2	12	340	7.64	5–11.4	23	301
10c	18.15	14.3–22.8	61	336	15.12	11.3–19.9	44	291
11a	4.82	2.8–8	15	311	7.53	4.8–11.4	21	279
11b	1.24	0.4–3.4	4	323	3.17	1.6–6.1	9	284
12	1.76	0.7–4	6	340	2.99	1.5–5.8	9	301
13a	87.50	83.4–90.7	294	336	89.90	85.8–93	267	297
13b	44.08	38.7–49.6	149	338	45.97	40.2–51.8	137	298
13c	10.06	7.2–13.9	34	338	12.75	9.3–17.2	38	298
14	0.29	0–1.9	1	340	0.00	0–1.6	0	301
15a	37.35	32.2–42.8	127	340	36.54	31.1–42.3	110	301
15b	12.65	9.4–16.8	43	340	14.95	11.2–19.6	45	301
16	78.55	73.7–82.8	260	331	81.03	75.9–85.3	235	290
17a	16.47	12.8–20.9	56	340	21.93	17.5–27.1	66	301
17b	1.18	0.4–3.2	4	340	1.66	0.6–4.1	5	301
18a	100.00	98.6–100	340	340	99.34	97.4–99.9	299	301
18b	26.47	21.9–31.6	90	340	27.57	22.7–33.1	83	301
18c	2.94	1.5–5.5	10	340	2.99	1.5–5.8	9	301
19	77.94	73.1–82.2	265	340	78.07	72.9–82.5	235	301
20	51.47	46–56.9	175	340	54.49	48.7–60.2	164	301

#### Landis item individual compliance

**Fig. 4:**
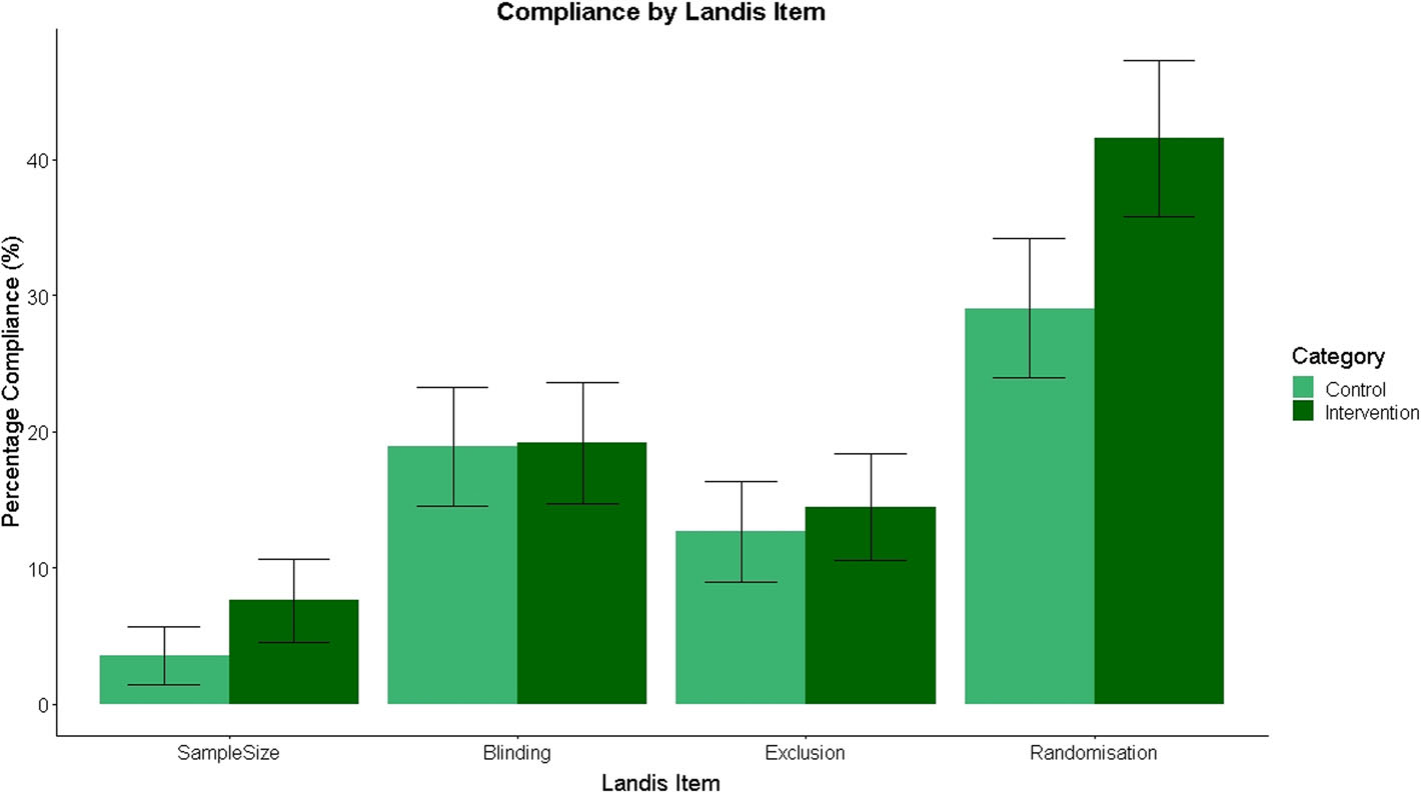
Landis 4 individual compliance; percentage compliance for each Landis criteria present in the ARRIVE guidelines with 95% confidence intervals

To determine if there had been any changes in individual Landis subitems, we investigated randomisation, blinding, reporting of a sample size calculation, and reporting of exclusions separately (Fig. 4). Although we did not analyse these comparisons using inferential statistics, there appears to be some improvements in reporting of randomisation and sample size calculations. 29.1% (91/313) of manuscripts in the control group reported whether or not random assignment occurred, compared to 41.5% (125/301) in the intervention group (Cohen’s H effect size = 0.26), while 3.5% (12/40) of control manuscripts reported sample size calculations compared to 7.6% (25/330) in the intervention group (Cohen’s H effect size = 0.18). For the reporting of animal exclusions, 12.6% (43/340) of manuscripts complied in the control group versus 14.5% (48/332) in the intervention group (Cohen’s H effect size = 0.05). Finally, 18.8% (63/334) and 19.2% (62/323) of manuscripts reported blinded outcome assessment in the control and intervention groups, respectively (Cohen’s H effect size = 0.01).

## Discussion

Requesting completion of an ARRIVE checklist at submission did not increase full adherence with the ARRIVE guidelines. Compliance with the operationalised ARRIVE checklist was poor overall, with no manuscripts in either group even approaching full compliance; the median compliance was less than 40%, equivalent to around 15 of 38 subitems; and the intervention only increased compliance with one subitem, reporting of animal husbandry conditions. There is considerable room for improvement, and this study shows that an editorial policy of making ARRIVE checklist completion “mandatory” without compliance checks has little or no impact.

There were some noticeable differences between the control and intervention group for some subitems e.g. the proportion of manuscripts compliant with *10b (Explain how the number of animals was arrived at. Provide details of any sample size calculation used)* was double the size in the intervention group compared to control (3.53% vs 7.53%; Cohen’s H effect size = 0.18). However, our power calculation determined that a meaningful effect would be substantially larger to justify the increased burden for authors associated with implementation by PLOS ONE.

It may be that simply requesting that authors complete checklist, without any additional editorial checks to determine whether the checklist is truly indicative of compliance, may not be enough to improve adherence to the ARRIVE guidelines. Adherence to the reporting guidelines within in the clinical literature such as CONSORT and STROBE have been widely assessed and may inform interventions to improve compliance with preclinical guidelines. Journal endorsement of these guidelines appear to have improved reporting quality [15, 18]; however, it is often unclear what actions journals take to promote adherence [17] and the extent of editorial involvement is likely to have an impact. Prior reports indicate that assessing compliance with reporting guidelines at the stage of peer review leads to a significant improvement of reporting quality [5]. Other approaches (e.g. actions on the part of funders or institutions) may also be beneficial, but a successful strategy is likely to be multi-dimensional. Further, the findings reported here and the limited agreement between outcome assessors both in this study and in the recent investigation of study quality following the introduction of a new editorial policy at *Nature* journals [12] suggests that an important part of guideline development should be refinement of the content, the number of items (with fewer generally being better), and the agreement between assessors. It may be that a more formal adoption of research improvement strategies, with an original focus on a smaller number of items judged by a stakeholder to be of greatest importance, will allow an incremental approach to enabling and measuring improvement.

Our findings are in line with prior reports that endorsement by editors and reviewers has not significantly improved reporting of ARRIVE quality items [3, 6]. We need therefore a better understanding of the barriers to implementing quality checklists for animal experiments. It has been suggested that requesting checklist adherence at the submission stage may be too late, given the observed correlation between reporting at the planning application stage and at the publication stage [19]. The PREPARE (Planning Research and Experimental Procedures on Animals: Recommendations for Excellence) guidelines [16] were published recently and may be a useful tool, in combination with the ARRIVE checklist, to promote a greater focus on experimental rigour at all stages of the research cycle.

Our results contrast with recent reports of improvement in quality following mandated checklist completion following a change in editorial policy at *Nature* journals [7, 12]. However, in both reports, study quality was retrospectively assessed in publications published prior to and after the introduction of the Nature quality checklist, which was established in 2015 as part of an organisation wide approach with substantial editorial involvement. In contrast, the current trial investigated an intervention targeted at selected manuscripts, without further editorial involvement.

Perhaps unsurprisingly, due to the additional time required for ARRIVE checklist requests, both the number of days manuscripts spent in the PLOS editorial office and the number of days from manuscript submission to AE assignment were found to be significantly longer in the intervention group. The editorial resource required to ensure that all accepted publications meet the requirements of the ARRIVE checklist is likely to be considerable, given that PLOS ONE is a high-volume publisher, with around 44,000 submissions per year. The most feasible and effective way to encourage compliance to the ARRIVE guidelines, or indeed any reporting guideline, remains to be determined, but an ongoing review of interventions to improve adherence to reporting guidelines may shed some light on this issue and direct future investigations [4].

Another consideration is the perceived clarity of the checklist to authors and reviewers. Although reviewer agreement was generally high, a few questions were less well understood by our outcome assessors which suggests the current guidelines may require clearer dissemination among the research community.

### Limitations

Due to modest sample sizes, we were unable to investigate whether the intervention was more successful in countries with high awareness and adoption of the ARRIVE guidelines such as the UK, where the ARRIVE guidelines were developed and where many institutions have endorsed them. Furthermore, we did not perform a power calculation for outcomes beyond our primary and main secondary outcomes and it is possible that this, coupled with stringent adjustments for multiplicity of testing in some instances, may have prevented us from detecting any significant differences.

Furthermore, our intervention only involved requests for authors to complete an ARRIVE checklist. PLOS ONE did not fully mandate checklist completion, as manuscripts without a checklist were still allowed to proceed through the trial. Furthermore, PLOS ONE did not evaluate the accuracy of the completed checklists against each manuscript. It is possible that further emphasis on evaluation and checklist adherence may result in an enhancement of study quality.

Our interpretation of compliance was also influenced by our operationalisation of the ARRIVE checklist used for outcome assessment. It was often difficult to determine how many of the details provided in the ARRIVE guidelines were sufficient for full compliance to that ARRIVE subitem.

There were unforeseen difficulties in attaining data for some outcomes, which meant that we could not assess all outcomes presented in our study protocol. This was most apparent for feasibility outcomes, where there were substantial deviations from our protocol. Furthermore, the project was subject to research waste due to overpowering our primary and secondary outcome measures. As manuscripts submitted to PLOS ONE as part of the study were treated differently, the existence of the study could have leaked to external sources; however, to the best of our knowledge, this was not the case.

Since the ARRIVE guidelines were developed by the NC3Rs, no individual employed by the NC3Rs was permitted to conduct any outcome assessment on the IICARus platform.

## Conclusions

Research must be described in sufficient detail to allow research users critically to appraise experimental design, to allow them to assess the validity of the findings presented. Replication studies require, for their design, full details of what was done. Transparency in the reporting of research is paramount. Manuscripts must therefore be described in enough detail for readers to understand the research methodology and make informed judgement of quality and risk of bias. At present, reporting quality is, on average, disappointingly poor. However, our findings show that simply requesting that researchers improve reporting is not effective. Editorial checks of compliance and further measures to mandate checklist completion may be required to see improvements in quality.

## Additional files

Additional file 1: Operationalised ARRIVE checklist for IICARus platform. (DOCX 62 kb)

Additional file 2: Manuscript error log. (DOCX 14 kb)

Additional file 3: **Table S1.** Kappa agreement for outcome assessors per operationalised checklist question. **Figure S1.** Distribution of kappa agreement between outcome assessors for operationalised checklist questions. (PDF 276 kb)

## Abbreviations

AE: Academic editor
ARRIVE: Animal Research: Reporting of In Vivo Experiments
IICARus: Intervention to Improve Compliance with the ARRIVE guidelines
IQR: Interquartile range
NC3Rs: National Centre for the Replacement, Refinement and Reduction of Animals in Research
PREPARE: Planning Research and Experimental Procedures on Animals: Recommendations for Excellence
