# Supplementary material for: A randomised controlled trial of an Intervention to Improve Compliance with the ARRIVE guidelines (IICARus)
Source: Res Integr Peer Rev. 2019 Jun 12;4:12. doi: 10.1186/s41073-019-0069-3 (PMC6560728; doi:10.1186/s41073-019-0069-3)
Supplement: Supplementary file 1 — Operationalised ARRIVE checklist for IICARus platform. (DOCX 62 kb) [file 41073_2019_69_MOESM1_ESM.docx]

Additional file 1: Operationalised ARRIVE checklist for IICARus platform; Q, question ID number in IICARus platform; questions not referred to explicitly in the ARRIVE guidelines (and removed from analysis) are shown in grey.

| **Section Title** | **Q** | **IICARus Question** | **ARRIVE Guidelines** | **Compliance** |
| --- | --- | --- | --- | --- |
| **TITLE/ABSTRACT** | | | |  |
| Title |  |  | **ARRIVE subitem 1** |  |
|  | **1.1.0** | Is the species of animal model studied reported in the title? | Provide as accurate and concise a description of the content of the article as possible. | Must answer **“Yes”** to **1.1.0**, **1.2.0**, and **1.3.0** |
|  | **1.2.0** | Is the biological mechanism, disease or pathophysiology studied, reported in the title? |  |  |
|  | **1.3.0** | Is the intervention or exposure reported in the title? |  |  |
| Abstract |  |  | **ARRIVE Item 2** |  |
|  | **2.1.0** | Is the objective or hypothesis reported in the abstract? | Provide an accurate summary of the background, research objectives, including details of the species or strain of animal used, key methods, principal findings and conclusions of the study. | Must answer **“Yes”** to **2.1.0**, **2.2.0**, **2.3.0**, **2.4.0**, **2.5.0**, **2.6.0,** and **2.7.0** |
|  | **2.2.0** | Is the biological mechanism, disease or pathophysiology studied, reported in the abstract? |  |  |
|  | **2.3.0** | Is the intervention or exposure reported in the abstract? |  |  |
|  | **2.4.0** | Is the species or strain studied stated anywhere in the abstract? |  |  |
|  | **2.5.0** | Are the key methods of the study briefly summarised? |  |  |
|  | **2.6.0** | Are the principal findings of the study briefly summarised? |  |  |
|  | **2.7.0** | Are the conclusions of the study briefly summarised? |  |  |
|  | **0.1.0** | What animal species are used in this research? | Not mentioned in ARRIVE guidelines (categorisation purposes only) | Not applicable |
|  | **0.2.0** | Does the manuscript include human study? | Not mentioned in ARRIVE guidelines (categorisation purposes only) | Not applicable |
| **INTRODUCTION** | | | |  |
| Background | |  | **ARRIVE Item 3a** |  |
|  | **3.1.0** | Do the authors refer to previous work in the literature relating to this field? | Include sufficient scientific background (including relevant references to previous work) to understand the motivation and context for the study, and explain the experimental approach and rationale. | Must answer **“Yes”** to **3.1** |
|  |  |  | **Arrive Item 3b** |  |
|  | **3.2.0** | Is a statement reported about the rationale for using that animal species or animal disease model to address the scientific objectives? | Explain how and why the animal species and model being used can address the scientific objectives and, where appropriate, the study’s relevance to human biology | Must answer **“Yes”** to **3.2.0** and **3.3.0** |
|  | **3.3.0** | If applicable to the research question, is there a statement describing the relevance of the study to human biology? |  |  |
| Objectives | |  | **ARRIVE Item 4** |  |
|  | **4.1.0** | Is the objective or hypothesis reported in the introduction? | Clearly describe the primary and any secondary objectives of the study, or specific hypotheses being tested. | Must answer **“Yes”** to **4.1.0** |
| **METHODS** | | | | |
| Ethical statement | |  | **ARRIVE Item 5** |  |
|  | **5.1.0** | Does the manuscript include an explicit statement of approval? | Indicate the nature of the ethical review permissions, relevant licences (e.g. Animal [Scientific Procedures] Act 1986), and national or institutional guidelines for the care and use of animals, that cover the research. | Must answer **“Yes”** to **5.1.0**, **5.2.0**, and **5.3.0** |
|  | **5.2.0** | Does the manuscript identify the committee(s) approving the study protocol? |  |  |
|  | **5.3.0** | Does the manuscript name the international, national or institutional guidelines followed? |  |  |
|  | **5.4.0** | Does the manuscript report a protocol / permit number? | Not mentioned in ARRIVE guidelines (removed from analysis) | Not applicable |
| Study Design | |  | **ARRIVE Item 6a** |  |
|  | **6.1.0** | Are the total number of experimental and control groups reported? | For each experiment, give brief details of the study design including: The number of experimental and control groups. | Must answer **“Yes”** to **6.1.0** |
|  |  |  | **ARRIVE Item 6c** |  |
|  | **6.2.0** | Is the experimental unit stated? | For each experiment, give brief details of the study design including: The experimental unit (e.g. a single animal, group or cage of animals). | Must answer **“Yes”** to **6.2.0** and **6.3.0** |
|  | **6.3.0** | If the experimental unit is not stated is it clear what it is? |  |  |
|  |  |  | **ARRIVE Item 6b** |  |
|  | **6.4.0** | Is randomisation reported? | For each experiment, give brief details of the study design including: Any steps taken to minimise the effects of subjective bias when allocating animals to treatment (e.g. randomisation procedure) and when assessing results (e.g. if done, describe who was blinded and when). | Must answer **“Yes”** to either **6.4.0** or **6.5.0** and either **6.6.0** or **6.7.0** |
|  | **6.5.0** | Does the manuscript include a statement about randomisation even if no randomisation was done? |  |  |
|  | **6.6.0** | Are assessors blinded for at least one of the outcomes measured? |  |  |
|  | **6.7.0** | Does the manuscript include a statement about blinding even if no done? |  |  |
| Experimental Procedures | |  | **ARRIVE Item 7a** |  |
|  | **7.1.1** | Vehicle(s) reported? | For each experiment and each experimental group, including controls, provide precise details of all procedures carried out. For example: a. How (e.g. drug formulation and dose, site and route of administration, anaesthesia and analgesia used [including monitoring], surgical procedure, method of euthanasia). Provide details of any specialist equipment used, including supplier(s). | Must answer **“Yes”** to **7.1.1**, **7.1.2**, **7.1.3**, **7.1.4**, **7.1.5**, **7.1.6**, **7.1.7**, **7.3.1**, **7.3.2**, **7.3.3**, **7.3.4**, **7.3.5**, **7.3.6**, **7.5.1**, **7.5.2**, **7.5.3**, **7.5.4**, **7.5.5**, **7.5.6**, **7.5.7**, **7.5.8**, **7.6.1**, and **7.6.2** |
|  | **7.1.2** | Vehicle volume(s) reported? |  |  |
|  | **7.1.3** | Intervention/exposure dose(s) reported? |  |  |
|  | **7.1.4** | Route(s) of administration reported? |  |  |
|  | **7.1.5** | Site(s) of administration reported? |  |  |
|  | **7.1.6** | Frequency of administration reported? |  |  |
|  | **7.1.7** | Supplier(s) reported? |  |  |
| Experimental Procedures (Control) | |  |  |  |
|  | **7.3.1** | Is the control reported? |  |  |
|  | **7.3.2** | Is the control dose or volume reported? |  |  |
|  | **7.3.3** | Is the control route reported? |  |  |
|  | **7.3.4** | Is the control site of administration reported? |  |  |
|  | **7.3.5** | Is the frequency of administration reported? |  |  |
|  | **7.3.6** | If a control (e.g. sham) surgical procedure was carried out do they describe the methods used? |  |  |
| Surgery and Anaesthesia |  |  |  |  |
|  | **7.5.1** | Is surgical anaesthesia use reported? |  |  |
|  | **7.5.2** | Is the anaesthesia route reported? |  |  |
|  | **7.5.3** | Is the anaesthetic reported? |  |  |
|  | **7.5.4** | Is the anaesthesia dose reported? |  |  |
|  | **7.5.5** | Are the methods used for surgical procedures clearly described? |  |  |
|  | **7.5.6** | Are the suppliers for any specialist surgical equipment reported? |  |  |
|  | **7.5.7** | Is the monitoring of at least one physiological parameters during surgical anaesthesia reported? |  |  |
|  | **7.5.8** | Is the use of an analgesic, or a reason why analgesic was not used, reported? |  |  |
| **Euthanasia** |  |  |  |  |
|  | **7.6.1** | Is euthanasia, sacrifice etc. reported? |  |  |
|  | **7.6.2** | Is the method of euthanasia reported? |  |  |
|  | **7.1.8** | If a surgical procedure was carried out was it part of model induction? | Not mentioned in ARRIVE guidelines (removed at validation phase) | Not applicable |
|  | **7.1.9** | If a surgical procedure was carried out was it part of either treatment or outcome measurement(s)? | Not mentioned in ARRIVE guidelines (removed from analysis) | Not applicable |
| Experimental Procedures | |  | **ARRIVE Item 7b** |  |
|  | **7.2.1** | Does the manuscript describe when the intervention/exposure group procedures were carried out? | For each experiment and each experimental group, including controls, provide precise details of all procedures carried out. For example: When (e.g. time of day). | Must answer **“Yes”** to **7.2.1** and **7.4.1** |
| Experimental Procedures (Control) | |  |  |  |
|  | **7.4.1** | Does the manuscript describe when the control/comparator intervention procedures were carried out? |  |  |
| Experimental Procedures | |  | **ARRIVE Item 7c** |  |
|  | **7.2.2** | Does the manuscript describe where the intervention/exposure group procedures were carried out? | For each experiment and each experimental group, including controls, provide precise details of all procedures carried out. For example: Where (e.g. home cage, laboratory, water maze). | Must answer **“Yes”** to **7.2.2** and **7.4.2** |
| Experimental Procedures (Control) | |  |  |  |
|  | **7.4.2** | Does the manuscript describe where the control/comparator intervention procedures were carried out? |  |  |
| Experimental Procedures | |  | **ARRIVE Item 7d** |  |
|  | **7.2.3** | Is any rationale for the use of the intervention/exposure reported? | For each experiment and each experimental group, including controls, provide precise details of all procedures carried out. For example: Why (e.g. rationale for choice of specific anaesthetic, route of administration, drug dose used). | Must answer **“Yes”** to **7.2.3** and **7.4.3** |
| Experimental Procedures (Control) | |  |  |  |
|  | **7.4.3** | Is any rationale for the use of the control/comparator group reported? |  |  |
| Experimental animals | |  | **ARRIVE Item 8a** |  |
|  | 8.1.0 | Is the animal species reported? | Provide details of the animals used, including species, strain, sex, developmental stage (e.g. mean or median age plus age range) and weight (e.g. mean or median weight plus weight range). | Must answer **“Yes”** to **8.1.0**, **8.2.0**, **8.3.0**, **8.4.0**, and **8.5.0** |
|  | 8.2.0 | Is the strain of the animals reported? |  |  |
|  | 8.3.0 | Is the sex of the animals reported? |  |  |
|  | 8.4.0 | Is the age of the animals reported? |  |  |
|  | 8.5.0 | Is the weight of the animals reported? |  |  |
|  |  |  | **ARRIVE Item 8b** |  |
|  | 8.6.0 | For studies using transgenic animals, do the authors report: 1) The genetic modification status (knockout, overexpression etc.), 2) The genotype (homozygous, heterozygous) and 3) The manipulated gene/s? | Provide further relevant information such as the source of animals, international strain nomenclature, genetic modification status (e.g. knock-out or transgenic), genotype, health/immune status, drug or test naïve, previous procedures, etc.  **ARRIVE Item 14** | Must answer **“Yes”** to **8.6.0** and **8.8.0** |
|  | 8.8.0 | Is the source/supplier of the animals reported? |  |  |
| **H**ousing | |  | **ARRIVE Item 9a** |  |
|  | 9.1.1 | Is the biosecurity level of the facility reported? | Housing (type of facility e.g. specific pathogen free [SPF]; type of cage or housing; bedding material; number of cage companions; tank shape and material etc. for fish). | Must answer **“Yes”** to **9.1.1**, **9.1.2**, **9.1.3**, **9.1.4**, and **9.2.4** |
|  | 9.1.2 | Is the type of cage or housing reported? |  |  |
|  | 9.1.3 | Is the bedding material reported? |  |  |
|  | 9.1.4 | Is the number of cage companions reported? |  |  |
|  | 9.2.4 | For experiments involving fish, are the tank dimensions or materials reported? |  |  |
| Husbandry | |  | **ARRIVE Item 9b** |  |
|  | 9.2.1 | Are the light/dark cycle conditions reported? | Husbandry conditions (e.g. breeding programme, light/dark cycle, temperature, quality of water etc for fish, type of food, access to food and water, environmental enrichment).  for fish, type of food, access to food and water, environmental enrichment). | Must answer **“Yes”** to any of **9.2.1**, **9.2.2**, **9.2.3**, **9.2.5**, or **9.2.6**, |
|  | 9.2.2 | Is the temperature reported? |  |  |
|  | 9.2.3 | For experiments involving fish, is the quality of the water reported? |  |  |
|  | 9.2.5 | Is the type of food provided reported? |  |  |
|  | 9.2.6 | Are the conditions around access to food reported? |  |  |
|  | 9.2.7 | Are the conditions around access to drinking water reported? |  |  |
|  | 9.2.8 | Is any environmental enrichment reported? |  |  |
| Welfare | |  | **ARRIVE Item 9c** |  |
|  | 9.3.1 | Have they reported any welfare assessment or intervention before, during, or after the experiment? | Welfare-related assessments and interventions that were carried out prior to, during, or after the experiment. | Must answer **“Yes”** to **9.3.1** |
| Sample size | |  | **ARRIVE Item 10a** |  |
|  | 10.1.0 | Is the total number of animal used for the experiment reported? | Specify the total number of animals used in each | Must answer **“Yes”** to **10.1.0** and **10.2.0** |
|  | 10.2.0 | Is the number of animals in each experimental group reported? |  |  |
|  |  |  | ARRIVE Item 10b |  |
|  | 10.3.0 | Is a sample size calculation reported? | Explain how the number of animals was arrived at. Provide details of any sample size calculation used. | Must answer **“Yes”** to **10.3.0** |
|  | 10.4.0 | Is the statistical method for the sample size calculation reported or any other explanation provided? |  |  |
|  |  |  | ARRIVE Item 10c |  |
|  | 10.5.0 | Is the number of independently replicated experiments reported? | Indicate the number of independent replications of each experiment, if relevant | Must answer **“Yes”** to **10.5.0** |
|  | 11.1.0 | Is allocation concealment reported? | Not mentioned in ARRIVE guidelines (removed from analysis) | Not applicable |
| Allocating animals to experimental groups | |  | **ARRIVE Item 11a** |  |
|  | 11.2.0 | Are the methods of allocation to group (i.e. randomisation, matching) described? | Give full details of how animals were allocated to experimental groups, including randomisation or matching if done. | Must answer **“Yes”** to **11.2.0** |
|  |  |  | ARRIVE Item 11b |  |
|  | 11.3.0 | Is the order in which animals receive treatments defined? | Describe the order in which the animals in the different experimental groups were treated and assessed. | **“Yes”** to **11.3.0** and **11.4.0** |
|  | 11.4.0 | Is the order in which outcomes are assessed in different animals reported? |  |  |
| Experimental outcomes | |  | ARRIVE Item 12 |  |
|  | 12.1.0 | Are outcomes reported identified as being either primary or secondary? | Clearly define the primary and secondary experimental outcomes assessed (e.g. cell death, molecular markers, behavioural changes).**12** | **“Yes”** to **12.1.0** and **12.2.0** |
|  | 12.2.0 | Is at least one outcome measure described? | Not mentioned in ARRIVE guidelines (removed from analysis) | Not applicable |
| **Statistical methods** | |  | ARRIVE Item 13a |  |
|  | 13.1.0 | Is at least one outcome measure associated with at least one statistical test? | Provide details of the statistical methods used for each analysis. | **“Yes”** to **13.1.0** |
|  |  |  | ARRIVE Item 13b |  |
|  | 13.2.0 | Is the unit of analysis for at least one tests explicitly specified? | Specify the unit of analysis for each dataset (e.g. single animal, group of animals, single neuron). | **“Yes”** to **13.2.0** |
|  |  |  | ARRIVE Item 13c |  |
|  | 13.3.0 | Does the publication include a method to assess whether the data meet the assumptions of the statistical tests used? | Describe any methods used to assess whether the data met the assumptions of the statistical approach. | **“Yes”** to **13.3.0** |
| **RESULTS** | | | |  |
| Numbers analysed | |  | ARRIVE Item 14 |  |
|  | 8.7.0 | Are the animals used in the study reported to be drug or test naïve prior to treatment or testing? | For each experimental group, report relevant characteristics and health status of animals (e.g. weight, microbiological status, and drug or test naïve) prior to treatment or testing. (This information can often be tabulated). | **“Yes”** to **8.7.0** and **8.9.0** |
|  | 8.9.0 | Is the health status of the animals reported? |  |  |
|  |  |  | ARRIVE Item 15a |  |
|  | 14.1.0 | Is the number of animals for each group reported for each analysis? | Report the number of animals in each group included in each analysis. Report absolute numbers (e.g. 10/20, not 50%2). | **“Yes”** to **14.1.0** |
|  |  |  | ARRIVE Item 15b |  |
|  | 14.2.0 | Are reasons for the exclusion of animals (for any outcome) given? | If any animals or data were not included in the analysis, explain why. | **“Yes”** to **14.2.0** |
|  |  |  | ARRIVE Item 16 |  |
|  | 15.1.0 | Are findings presented with a measure of precision? | Report the results for each analysis carried out, with a measure of precision (e.g. standard error or confidence interval). | **“Yes”** to **15.1.0** and **15.2.0** |
|  | 15.2.0 | Is the measure of precision defined? |  |  |
|  |  |  | ARRIVE Item 17a |  |
|  | 16.1.0 | Is there a statement indicating whether or not adverse events occurred for at least one experimental group? | Give details of all important adverse events in each experimental group. | **“Yes”** to **16.1.0** |
|  |  |  | ARRIVE Item 17b |  |
|  | 16.2.0 | Are any modifications to the experimental design to reduced adverse effects reported? | Describe any modifications to the experimental protocols made to reduce adverse events. | **“Yes”** to **16.2.0** |
| **DISCUSSION** | |  |  |  |
| Interpretation/ scientific implications | |  | ARRIVE Item 18a |  |
|  | 17.1.0 | Are the results interpreted in the context of the study hypothesis or objectives? | Interpret the results, taking into account the study objectives and hypotheses, current theory and other relevant studies in the literature. | **“Yes”** to **17.1.0** and **17.2.0** |
|  | 17.2.0 | Are the results interpreted in the context of other studies in the literature? |  |  |
|  |  |  | ARRIVE Item 18b |  |
|  | 17.3.0 | Are the limitations of the study design and/or execution discussed? | Comment on the study limitations including any potential sources of bias, any limitations of the animal model, and the imprecision associated with the results. | **“Yes”** to **17.3.0** |
|  |  |  | ARRIVE Item 18c |  |
|  | 17.4.0 | Are any implications of the experimental methods or findings for the replacement, refinement or reduction (the 3Rs) of the use of animals in research discussed? | Describe any implications of your experimental methods or findings for the replacement, refinement or reduction (the 3Rs) of the use of animals in research. | **“Yes”** to **17.4.0** |
| Generalisability/ translation | |  | ARRIVE Item 19 |  |
|  | 18.1.0 | Is there a statement about how the findings of this study might translate to other species or systems, such as any relevance to human biology? | Comment on whether, and how, the findings of this study are likely to translate to other species or systems, including any relevance to human biology. | **“Yes”** to **18.1.0** |
| Funding | |  | ARRIVE Item 20 |  |
|  | 19.1.0 | Do the authors report funding source(s)? | List all funding sources (including grant number) and the role of the funder(s) in the study. | **“Yes”** to **19.1.0**, **19.2.0**, and **19.3.0** |
|  | 19.2.0 | Do the authors include the grant number (grant #)? |  |  |
|  | 19.3.0 | Has the role of the funders been reported? |  |  |
|  | 19.4.0 | Is there a statement of competing/conflict of interests? | Not mentioned in ARRIVE guidelines (removed from analysis) | Not applicable |
