# Supplementary material for: A randomised controlled trial of an Intervention to Improve Compliance with the ARRIVE guidelines (IICARus)
Source: Res Integr Peer Rev. 2019 Jun 12;4:12. doi: 10.1186/s41073-019-0069-3 (PMC6560728; doi:10.1186/s41073-019-0069-3)
Supplement: Supplementary file 2 — Manuscript error log. (DOCX 14 kb) [file 41073_2019_69_MOESM2_ESM.docx]

Additional file 2

| Reason for Error | Number of Manuscripts |
| --- | --- |
| No comment added in system (unknown source of error) | 2 |
| Appeal/resubmission from before trial start date | 28 |
| Incorrectly deleted from trial | 2 |
| Selected for intervention but email request not sent | 2 |
| Manuscript no longer in Editorial Manager (removed by author) | 32 |
| Not an *in vivo* animal study | 77 |
| Randomised after initial failed technical check email was sent | 1 |
| Randomised twice | 9 |
| Grand Total | **153** |
