# Supplementary material for: A randomised controlled trial of an Intervention to Improve Compliance with the ARRIVE guidelines (IICARus)
Source: Res Integr Peer Rev. 2019 Jun 12;4:12. doi: 10.1186/s41073-019-0069-3 (PMC6560728; doi:10.1186/s41073-019-0069-3)

Supplementary Table 1: Kappa agreement for outcome assessors per operationalised checklist question; Question Number, operationalised checklist number on IICARus platform; CI, confidence interval; Kappa Estimate, unweighted Cohen's kappa; NA for ARRIVE Item indicates that the information required for this questions is not a requirement of the ARRIVE guidelines

Question Number	ARRIVE Item	Lower CI	Upper CI	Kappa Estimate	IICARUs Question
1.1	1	0.862	0.929	0.896	Is the species of animal model studied reported in the title?
1.2	1	0.094	0.388	0.241	Is the biological mechanism, disease or pathophysiology studied, reported in the title?
1.3	1	0.213	0.345	0.279	Is the intervention or exposure reported in the title?
2.1	2	0.11	0.297	0.204	Is the objective or hypothesis given in the abstract?
2.2	2	-0.105	0.318	0.106	Is the biological mechanism, disease or pathophysiology studied, reported in the abstract?
2.3	2	0.231	0.402	0.317	Is the intervention or exposure reported in the abstract?
2.4	2	0.553	0.73	0.642	Is the species or strain studied stated anywhere in the abstract?
3.1	3	-0.009	0	-0.004	Do the authors refer to previous work in the literature relating to this field?
3.2	3	0.447	0.581	0.514	Is a statement reported about the rationale for using that animal species or animal disease model to address the scientific objectives?
3.3	3	0.282	0.433	0.358	If applicable to the research question, is there a statement describing the relevance of the study to human biology?
4.1	4	0.043	0.231	0.137	Is the objective or hypothesis given in the introduction?
5.1	5	0.043	0.458	0.25	Does the manuscript include an explicit statement of approval?
5.2	5	0.127	0.403	0.265	Does the manuscript identify the committee(s) approving the study protocol?
5.3	5	0.707	0.817	0.762	Does the manuscript name the international, national or institutional guidelines followed?
5.4	NA	0.812	0.893	0.853	Does the manuscript report a protocol / permit number?
6.1	6	0.154	0.308	0.231	Are the total number of experimental and control groups reported?
6.2	6	0.034	0.203	0.119	Is the experimental unit stated?
6.3	6	0.08	0.232	0.156	If the experimental unit is not stated is it clear what it is?
6.4	6	0.633	0.735	0.684	Is randomisation reported to assign animals to experimental group?
6.5	6	0.509	0.623	0.566	If randomisation was NOT done, does the paper mention randomisation at all?
6.6	6	0.663	0.791	0.727	Are assessors blinded for at least one of the outcomes measured?
6.7	6	0.515	0.662	0.588	Does the manuscript include a statement about blinding even if no blinding was done?
7.1.8	NA	0.529	0.644	0.587	If a surgical procedure was carried out was it part of model induction?
7.1.1	7	0.421	0.529	0.475	Vehicle(s) reported?
7.1.2	7	0.313	0.426	0.369	Vehicle volume(s) reported?
7.1.3	7	0.576	0.696	0.636	Intervention/exposure dose(s) reported?

7.1.4	7	0.601	0.716	0.658	Route(s) of administration reported?
7.1.5	7	0.491	0.606	0.549	Site(s) of administration reported?
7.1.6	7	0.386	0.508	0.447	Frequency of administration reported?
7.1.7	7	0.448	0.563	0.505	Supplier(s) reported?
7.1.9	NA	0.516	0.633	0.575	If a surgical procedure was carried out was it part of either treatment or outcome measurement(s)?
7.2.1	7	0.257	0.378	0.317	Does the manuscript describe when the intervention/exposure group procedures were carried out?
7.2.2	7	0.244	0.38	0.312	Does the manuscript describe where the intervention/exposure group procedures were carried out?
7.2.3	7	0.166	0.295	0.231	Is any rationale for the use of the intervention/exposure group reported?
7.3.1	7	0.268	0.401	0.335	Is the control reported?
7.3.2	7	0.423	0.53	0.477	Is the control dose or volume reported?
7.3.3	7	0.483	0.588	0.535	Is the control route reported?
7.3.4	7	0.436	0.543	0.489	Is the control site of administration reported?
7.3.5	7	0.395	0.503	0.449	Is the frequency of administration reported?
7.4.1	7	0.314	0.426	0.37	Does the manuscript describe when any control /comparator interventions were carried out?
7.4.2	7	0.359	0.485	0.422	Does the manuscript describe where any control/comparator interventions were carried out?
7.4.3	7	0.3	0.432	0.366	Is any rationale for the use of the control/comparator group reported?
7.3.6	7	0.35	0.54	0.445	If a control surgical procedure (sham) was carried out, do they describe the methods used?
7.5.1	7	0.581	0.692	0.636	Is surgical anaesthesia use reported?
7.5.2	7	0.602	0.71	0.656	Is the anaesthesia route reported?
7.5.3	7	0.658	0.766	0.712	Is the anaesthetic reported?
7.5.4	7	0.623	0.731	0.677	Is the anaesthesia dose reported?
7.5.7	7	0.618	0.729	0.673	Is the monitoring of at least one physiological parameters during surgical anaesthesia reported?
7.6.1	7	0.633	0.771	0.702	Is euthanasia, sacrifice etc. reported?
7.6.2	7	0.611	0.712	0.662	Is the method of euthanasia reported?
8.1	7	0	0	0	Is the animal species reported?
8.2	7	0.219	0.518	0.368	Is the strain of the animals reported?
8.3	7	0.64	0.754	0.697	Is the sex of the animals reported?
8.4	7	0.62	0.713	0.666	Is the age of the animals reported?
8.5	7	0.645	0.746	0.695	Is the weight of the animals reported?
8.6	8	0.472	0.575	0.524	For studies using transgenic animals, do the authors report: 1) The genetic modification status (knockout, overexpression etc.), 2) The genotype (homozygous, heterozygous) and 3) The manipulated gene/s?
8.7	14	0.07	0.406	0.238	Are the animals used in the study reported to be drug or test naive prior to treatment or testing?
8.8	8	0.586	0.712	0.649	Is the source/supplier of the animals reported?
8.9	14	0.345	0.59	0.467	Is the health status of the animals reported?
9.1.1	9	0.634	0.767	0.7	Is the biosecurity level of the facility reported?
9.1.2	9	0.609	0.736	0.672	Is the type of cage or housing reported?
9.1.3	9	0.704	0.845	0.774	Is the bedding material reported?

9.1.4	9	0.735	0.842	0.789	Is the number of cage companions reported?
9.2.2	9	0.855	0.923	0.889	Are the light/dark cycle conditions reported?
9.2.3	9	0.788	0.879	0.833	Is the temperature reported?
9.2.4	9	0.587	0.806	0.696	For experiments involving fish, is the quality of the water reported?
9.2.1	9	0.708	0.903	0.806	For experiments involving fish, are the tank dimensions or materials reported?
9.2.5	9	0.711	0.811	0.761	Is the type of food provided reported?
9.2.6	9	0.776	0.86	0.818	Are the conditions around access to food reported?
9.2.7	9	0.802	0.879	0.84	Are the conditions around access to drinking water reported?
9.2.8	9	0.488	0.728	0.608	Is any environmental enrichment reported?
9.3.1	9	0.401	0.564	0.482	Have they reported any welfare assessment or intervention before, during, or after the experiment?
10.1	10	0.496	0.622	0.559	Is the total number of animals used in the study reported?
10.2	10	0.396	0.506	0.451	Is the number of animals in each experimental group reported?
10.3	10	0.534	0.776	0.655	Is a sample size calculation reported?
10.4	10	0.036	0.199	0.117	Is the basis for the sample size calculations (effect size, variance, power) presented? If response to 10.3 is "No", select NA
10.5	10	0.3	0.431	0.365	Is the number of independent replications of experiments reported?
11.1	NA	0.094	0.309	0.202	Is allocation concealment reported?
11.2	11	0.178	0.349	0.264	Are the methods of allocation to group (i.e. randomisation, matching) described?
11.4	11	0.288	0.449	0.369	Is the order in which animals receive treatments defined?
11.5	11	0.06	0.24	0.15	Is the order in which outcomes are assessed in different animals reported?
12.1	12	-0.043	0.114	0.036	Are reported outcomes identified as primary or secondary?
12.2	NA	-0.061	0.137	0.038	Is at least one outcome measure described?
13.1	13	0.242	0.432	0.337	Is at least one outcome measure associated with at least one statistical test?
13.2	13	-0.101	0.043	-0.029	Is the unit of analysis for at least one test explicitly specified?
13.3	13	0.23	0.421	0.325	Does the publication include a method to assess whether the data meet the assumptions of the statistical tests used?
14.1	15	0.218	0.331	0.274	Is the number of animals for each group reported for each analysis?
14.2	15	0.354	0.553	0.454	Are the reasons for the exclusion of animals (for any outcome) given?
15.1	16	0.212	0.413	0.312	Are findings presented with a measure of precision?
15.2	16	0.379	0.512	0.446	Is the measure of precision defined?
16.1	17	0.483	0.651	0.567	Is there a statement indicating whether or not adverse events occurred for at least one experimental group?
16.2	17	-0.085	0.202	0.059	Does the paper describe any refinements to the experimental design to reduce adverse events?
17.1	18	-0.017	-0.005	-0.011	Are the results interpreted in the context of the study hypothesis or objectives?

17.2	18	-0.02	-0.004	-0.012	Are the results interpreted in the context of other studies in the literature?
17.3	18	0.482	0.625	0.554	Are the limitations of the study design and/or execution discussed?
17.4	18	0.054	0.339	0.197	Are any implications of the experimental methods or findings for the for the replacement, refinement or reduction (the 3Rs) of the use of animals in research discussed?
18.1	19	0.316	0.474	0.395	Is there a statement about how the findings of this study might translate to other species or systems, such as any relevance to human biology?
19.1	20	0.284	0.568	0.426	Do the authors report funding source(s)?
19.2	20	0.681	0.794	0.738	Do the authors include the grant number (grant #)?
19.3	20	0.85	0.919	0.885	Has the role of the funders been reported?
19.4	NA	0.006	0.277	0.141	Is there a statement of competing or conflicting interests?
2.5	2	0.254	0.43	0.342	Are the key methods of the study briefly summarised?
2.6	2	-0.017	-0.004	-0.011	Are the principal findings of the study briefly summarised?
2.7	2	0.196	0.591	0.394	Are the conclusions of the study briefly summarised?
7.5.5	7	0.563	0.672	0.617	Are the methods used for surgical procedures clearly described?
7.5.6	7	0.522	0.639	0.581	Are the suppliers for any specialist surgical equipment reported?
7.5.8	7	0.602	0.716	0.659	Is the use of an analgesic, or a reason why analgesic was not used, reported?
0.2	NA	0.537	0.735	0.636	Does the manuscript include human study?

Supplementary Figure 1: Distribution of kappa agreement between outcome assessors for operationalised checklist questions

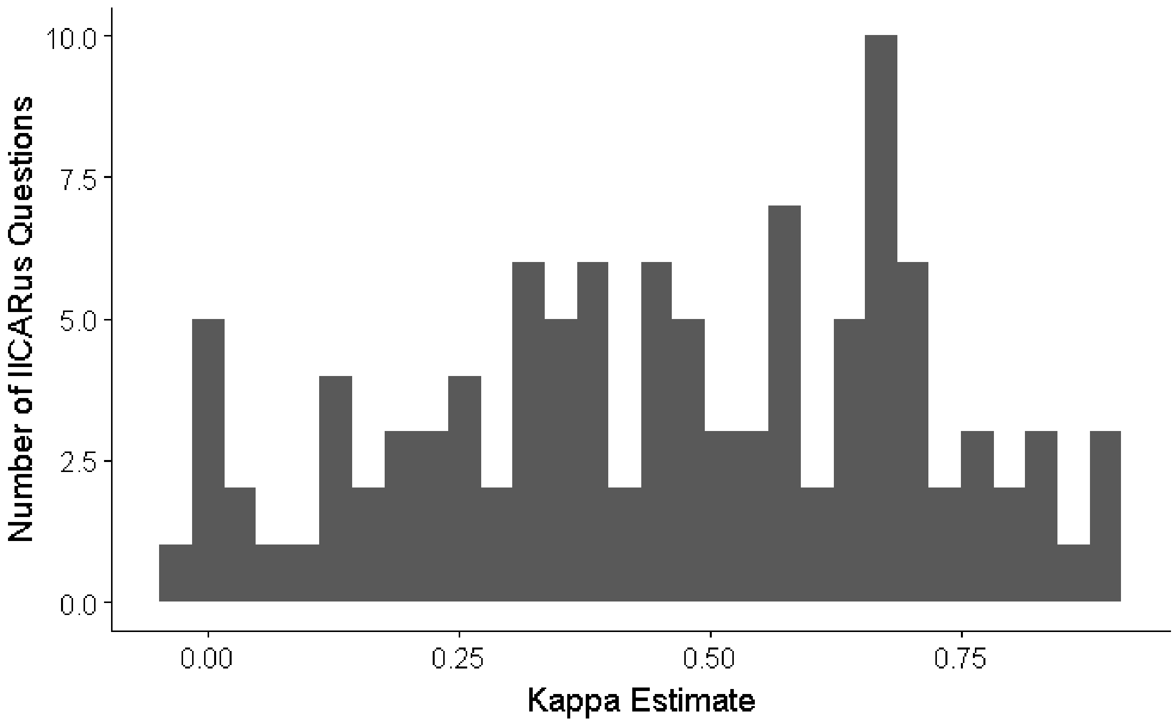

Supplement: Supplementary file 3 — Table S1. Kappa agreement for outcome assessors per operationalised checklist question. Figure S1. Distribution of kappa agreement between outcome assessors for operationalised checklist questions. (PDF 276 kb) [file 41073_2019_69_MOESM3_ESM.pdf]
